# Congenital Agenesis of Right Internal Carotid Artery: A Report of Two Cases

**DOI:** 10.5334/jbr-btr.1015

**Published:** 2016-03-15

**Authors:** Aysegul Sagir Kahraman, Bayram Kahraman, Zeynep Maras Ozdemir, Metin Dogan, Mehmet Kaya, Cemile Ayse Gormeli, Mehmet Akif Durak

**Affiliations:** 1 Department of Radiology, Inonu University School of Medicine, Malatya, Turkey; 2 Department of Radiology, Private Malatya Hospital, Malatya, Turkey; 3 Department of Neurosurgery, Inonu University School of Medicine, Malatya, Turkey

**Keywords:** Internal carotid artery, Agenesis, CT Imaging, MR Imaging, MR Angiography

## Abstract

Congenital unilateral agenesis of the internal carotid artery (ICA) is a rare anomaly. Due to proper sufficient collateral circulation via the circle of Willis most cases are asymptomatic, but patients can also present with ischemic or hemorrhagic cerebrovascular insults. The absence of the bony carotid canal is essential to differentiate this anomaly from chronic ICA occlusion. Awareness of this situation by clinicians and radiologists is essential because these patients have an increased incidence of various intracranial pathologies. We report two cases of this rare developmental congenital abnormality occurring in two young patients and describe the presentation, diagnosis, determined developmental causes, imaging findings, and complications.

## Introduction

The anomalies of the internal carotid artery (ICA) related to developmental defects can be categorized as follows: agenesis (absence of both ICA and bony carotid canal); aplasia (remnant of the ICA and bony carotid canal persist); or hypoplasia (one of the ICA is small but is regular in structure, as well as carotid canal) [4]. These are infrequent congenital anomalies of which the frequency has been reported to be less than 0.01 % [10]. Most cases of ICA agenesis are asymptomatic due to sufficient collateral circulation and it is usually an incidental finding on head and the neck imaging by color Doppler ultrasonography, computed tomography (CT), or magnetic resonance imaging (MRI).

In this article we report two cases of right internal carotid agenesis in a young female patient with nonspecific neurological findings and in a young male patient who presented with both nonspecific and specific neurological findings.

## Case 1

**Figure 1:**
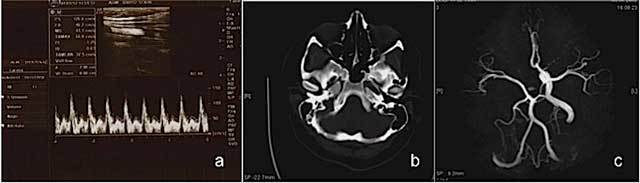
Ultrasound and Color Doppler images reveal right ICA agenesis and externalization flow pattern of right CCA (a); an axial CT image at bone window reveals absent right bony carotid canal (b); MR angiography image shows right ICA agenesis and anomalous origin of ophthalmic artery (c) in Case 1.

A 19-year-old female patient with a few days history of severe headache, dizziness, nausea, vomiting and syncope was admitted for cranial CT examination. Her physical and neurological examinations were normal and her medical history was unremarkable. The color Doppler sonography revealed absence of the right ICA and a changing flow pattern of common carotid artery, from a low peripheral resistance pattern proximally into a high peripheral resistance pattern similar to that seen in the external carotid artery distally (Figure 1a). The brain CT showed absent right ICA and bony carotid canal without any intraparanchymal pathology (Figure 1b). MRI and MR angiography findings showed right ICA agenesis and as well as anomalous origin of the ophthalmic artery derived from right middle cerebral artery (MCA). The aortic arch and major aortic branches were normal (Figure 1c).

## Case 2

**Figure 2:**
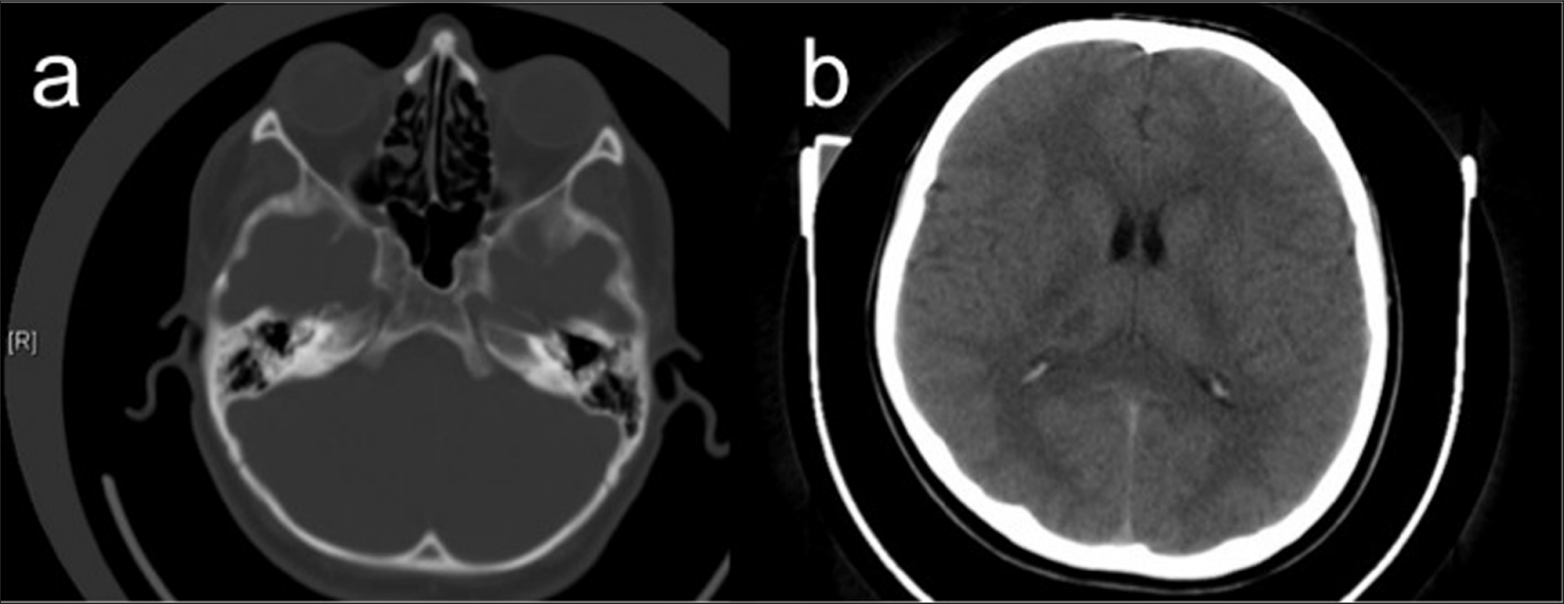
An axial CT image at bone window reveals absent right bony carotid canal (a); cranial CT image reveals hypodense lesion at right subthalamic region about 1 cm in diameter (b) in Case 2.

**Figure 3:**
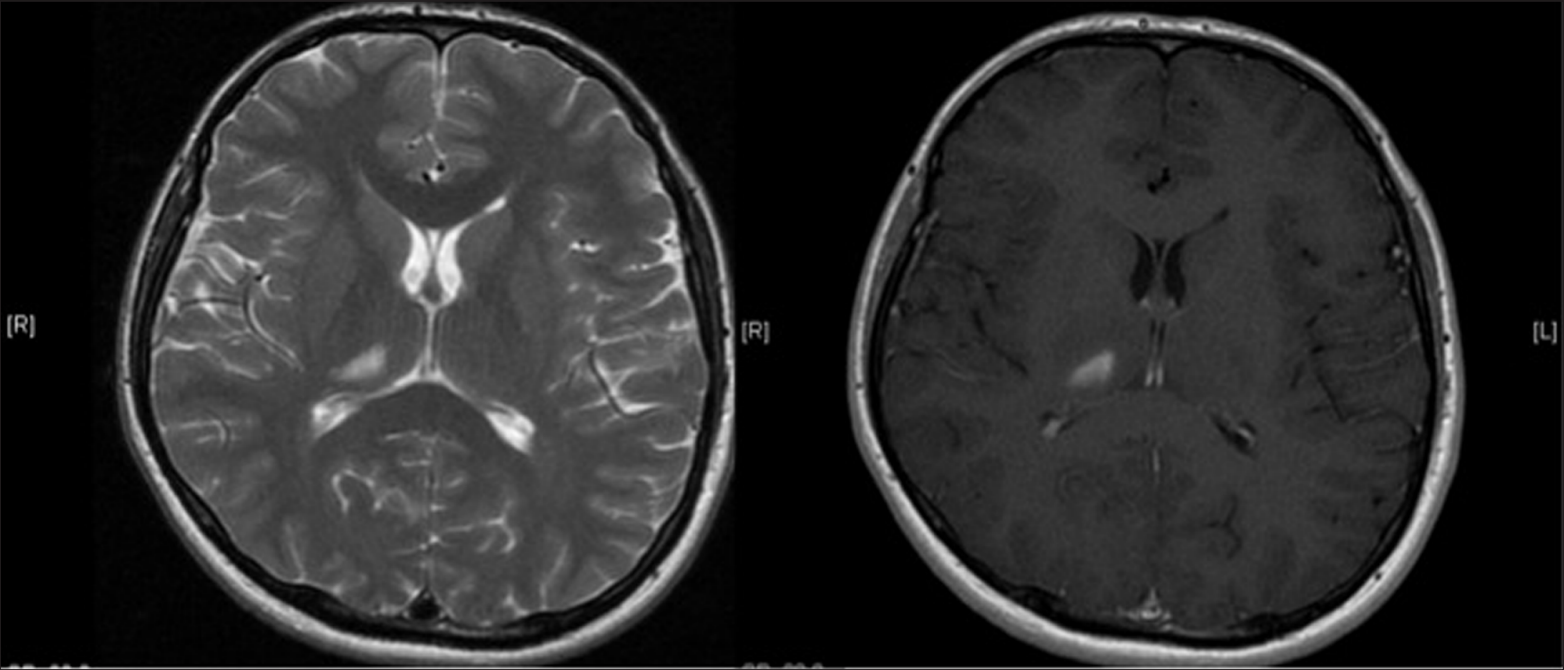
An axial T2W and enhanced T1W images show lesion consistent with subacute infarction (Case 2).

A 27-year-old male patient with an unremarkable medical history presented with acute onset of severe headache, dizziness and disequilibrium, and left sided weakness. Unenhanced CT revealed a hypodense lesion in the right thalamic and subthalamic region about 1 cm in diameter and an absent right ICA and bony carotid canal (Figure 2). On MRI, a well defined T2-hyperintense, T1-hypointense, contrast-enhancing lesion was seen in the right thalamus and subthalamic region consistent with sub-acute infarction (Figure 3). Cranial and cervical MR angiography showed right ICA agenesis and anomalous origin of the ophthalmic artery derived from right MCA. The aortic arch and major aortic branches were normal.

## Discussion

Agenesis of ICA firstly reported by Tode in 1787 [8] after postmortem examination. Thereafter Verbiest [9] reported this anomaly angiographically in 1954. According to the classification of developmental anomalies of ICA, our cases can particularly be categorized as agenesis. This condition has been occurred more often at the left side by 3:1 ratio [3].

The reported frequency is low, but as most patients are asymptomatic, the condition probably underreported. The vast majority of reported asymptomatic cases with ICA agenesis are one-sided, although there are scarce papers of asymptomatic cases of bilateral ICA agenesis [7]. Differential diagnoses, such as total occlusion or dissection, must be excluded by inspecting the bony carotid canal on CT, as the demonstration of a normal bony carotid canal rules out developmental ICA anomalies [6].

The agenesis of ICA is associated with a higher incidence of intracranial aneurysms, transsphenoidal encephaloceles, and an extensive rete mirabilis (vascular network interrupting the continuity of an artery or vein in the tissue) in the cranial base [1,6]. The clinical symptoms may be related with associated vascular insufficiency and/or intracranial ischemia due to changes in collateral flow. Cases may present with recurrent headaches, blurring vision, loss of audition, hemi-paresis with or without cranial nerve palsy [2,3,5,6]. Patients may also present with subarachnoid hemorrhage as a complication of an associated aneurysm. Intracranial aneurysms are found in approximately 25% of patients of symptomatic ICA agenesis presented with all intracranial hemorrhagic manifestations [6]. In cases of unilateral or bilateral ICA agenesis, the reported associated structural anomalies are agenesia of the corpus callosum and persistent cavum verge, arachnoidal cyst, anomaly of the basilar artery, olivopontocerebellar atrophy, hypopituitarism neurofibromatosis, meningocele, coarctation of the aorta, and cardiac abnormalities [3,6]. Embryologically, the primitive internal carotid arteries (ICAs) originate from terminal segments of the dorsal aorta and third aortic arch arteries at around the 3 mm fetal stage (24th day of embryogenesis), with complete development of the ICA by six weeks [2,10]. The development of the carotid channel is directly associated with the development of the ICA. At the fifth to sixth weeks of fetal life, the skull base begins to develop. If the primitive internal carotid arteries fail to develop before 3–5 weeks of fetal life, the bony carotid canal cannot develop [3]. In both of our cases, lack of both the ICA and the carotid canal was compatible with an incident having eventuated before three to five weeks of fetal life.

The ophthalmic artery has a sophisticated embryogenesis, which is closely related to with the development of the internal carotid artery [7]. According to Padget, when the embryo is 4–8 mm, two main primitive arteries supply the orbit [2]. The dorsal ophthalmic artery ascending from the cavernous part of the internal carotid artery inserts the orbit across the superior orbital fissure [7]. The ventral ophthalmic artery, originating at that time from the anterior cerebral artery, passes through the optic canal and supplies the optic tract [5]. The ventral ophthalmic artery usually persists to constitute the future ophthalmic artery. The adult form of the ophthalmic artery is identified at the 40 mm stage of the embryo [7]. The ophthalmic artery mostly originates from the intradural portion of the ICA just distal to the dural ring and passes through the optic canal. However, it can also infrequently ascend from the middle meningeal artery, anterior cerebral artery, accessory meningeal artery, basilar artery, middle cerebral artery, the posterior communicating artery, anterior deep temporal artery and the external carotid artery [5]. In our both patients, the anomalous ophthalmic artery originated from the middle cerebral artery.

In patients with unilateral or bilateral ICA agenesis three types of collateral pathway have been described. The most-common type of these collateral circulations is the fetal type, in which the ipsilateral anterior cerebral artery and the ipsilateral medial cerebral artery are supplied by the normal contralateral ICA through the anterior communicating artery and by the basilar artery through an ipsilateral dominant posterior communicating artery respectively [2]. In the second type, termed the adult form, the contralateral anterior cerebral artery supplies the ipsilateral anterior cerebral and middle cerebral arteries of the affected side via the anterior communicating artery [4]. In the rarest and third type, arterial blood supply is maintained through transcranial anastomosis developed from the external carotid system, from the contralateral ICA, or from certain primitive vessels such as persistent hypoglossal arteries, persistent tympanic or stapedial arteries, trigeminal arteries [6,10]. In our patients, the fetal form of collateralisation was observed in patient 1 and the adult form in patient 2.

## Conclusion

Congenital agenesis of the ICA is a rare, usually asymptomatic, vascular anomaly. Although most patients are asymptomatic rarely, they can be presented with some neurological disorders including intracranial hemorrhage or ischemia. To our knowledge an intracranial ischemia as in our second case is a very rare complication. The goals of imaging when suspecting ICA agenesis are: confirming the diagnosis by evaluating the bony carotid canal, identifying the type of the collateral circulation, assessing the presence of associated anomalies and ruling out acute complications including intracranial hemorrhage and ischemic lesions. MRI and MR angiography coupled with CT and color Doppler sonography allow accurate diagnosis. It is also beneficial for the educating of the patients and families about the benign feature of the disease and the oncoming implications, as well as the potential complications.

## Competing Interests

The authors declare that they have no competing interests.
